# The outcome of acute leukemia patients with SET-NUP214 fusion after allogeneic stem cell transplantation

**DOI:** 10.3389/fonc.2023.1256043

**Published:** 2023-10-13

**Authors:** Yuyan Shen, Donglin Yang, Rongli Zhang, Xin Chen, Qiaoling Ma, Jialin Wei, Weihua Zhai, Aiming Pang, Yi He, Erlie Jiang, Sizhou Feng

**Affiliations:** ^1^ State Key Laboratory of Experimental Hematology, National Clinical Research Center for Blood Diseases, Haihe Laboratory of Cell Ecosystem, Institute of Hematology and Blood Diseases Hospital, Chinese Academy of Medical Sciences and Peking Union Medical College, Tianjin, China; ^2^ Tianjin Institutes of Health Science, Tianjin, China

**Keywords:** acute myeloid leukemia, acute T lymphoblastic leukemia, allogeneic stem cell transplanation, SET-CAN/NUP214 fusion, del(9q)

## Abstract

SET-NUP214 fusion gene, also known as TAF-1-CAN and SET-CAN, is observed in acute myeloid leukemia (AML) and T-cell lymphoblastic leukemia (T-ALL). SET-NUP214 fusion in T-cell lymphoblastic leukemia is associated with chemotherapy resistance, but the prognosis of patients with AML with SET-NUP214 has rarely been reported. In the present study, we retrospectively analyzed all patients with acute leukemia including AML and T-ALL patients with SET-NUP214 fusion who underwent allogeneic stem cell transplantation (alloHSCT) in our center from July 2017 to November 2022. Of the total 11 patients, 5 patients were diagnosed with AML and 6 patients were diagnosed with T-ALL *de novo*. All patients received myeloablative regimens in CR1, and there were three (60%) AML patients who relapsed post-alloHSCT and three T-ALL (50%) patients who relapsed post-alloHSCT. Only one patient with AML who relapsed post-alloHSCT responded to subsequent chemotherapy plus donor lymphocyte infusion and survived the last follow-up. The estimated 1-year overall survival and 3-year overall survival for all these 11 patients were 69.3% and 38.5%, respectively. The estimated 1-year leukemia-free survival and 3-year leukemia-free survival for all patients were 69.3% and 38.5%, respectively. The research shows a high incidence of relapse for patients with acute leukemia with the SET-NUP214 fusion gene, even after alloHSCT. More clinical trials or research with larger samples are urgently needed for this group of patients.

## Introduction

The SET-NUP214 fusion gene, which results from either cryptic t(9;9)(q34;q34) or del(9)(q34.11q34.13), was first described in a patient with acute undifferentiated leukemia (1). Subsequently, several researchers reported that the fusion gene was also found in patients with acute myeloid leukemia (AML) and T-cell lymphoblastic leukemia (T-ALL) (2–4). SET-NUP214 fusion in patients with T-ALL is associated with corticosteroid/chemotherapy resistance but may respond to hematopoietic stem cell transplantation (HSCT) (5). The impact of SET-NUP214 fusion on patients with AML has rarely been reported. Due to the limited occurrence of SET-NUP214, with the reporting occurrence varying from 4.9 to 6% in T-ALL (5, 6), the outcome of acute leukemia patients with SET-NUP214 after hematopoietic stem cell transplantation has rarely been reported. In the present study, we retrospectively analyzed 11 acute leukemia patients with positive SET-NUP214 fusion gene who underwent allogeneic stem cell transplantation in our center from July 2017 to Nov 2022. These patients’ prognoses were rather poor despite the utilization of myeloablative conditioning.

## Methods

### Patient characteristics

Between July 2017 and Nov 2022, 11 patients with acute leukemia presenting SET-NUP214 who underwent allogeneic stem cell transplantation (alloHSCT) were enrolled in this study. Patients had a median age of 29 years (ranging from 17-43 years) at transplant. Seven of the patients were men and four were women. Five patients were diagnosed with AML, and six patients were diagnosed with T-ALL de nova, according to the fifth edition of the WHO classification. Of the 11 patients, 6 achieved hematological complete remission after one cycle of induction chemotherapy. Only 1 of the 11 patients achieved molecular complete remission after one cycle of induction chemotherapy. The median cycle of chemotherapy to achieve CR1 was one (ranging from one to four). All the patients received a median of four cycles (ranging from three to six) of chemotherapy before alloHSCT. The median time from diagnosis to transplant was 6 months (ranging from 6 to 8 months). All the patients underwent alloHSCT in CR1, but only three patients achieved negative SET-NUP214 fusion gene before alloHSCT. One patient with T-ALL presented with central nervous system leukemia involvement before HSCT and was in remission at transplant. The details of the patients before alloHSCT are summarized in **Tables 1**, **2**.

**Table 1:** Patient and transplant related characteristics.

Median patient age, years (range)	29(17-43)
Patient sex,n	
Male	7
Female	4
Diagnosis	
T-ALL	6
AML	
AML-M5	1
AML without maturation	3
AML not specified	1
Mean WBC count at diagnosis (×10^9^/L) (range)	
AML	16.42 (1.63-50.00)
T-ALL	55.96 (0.26-223)
Cycles of chemotherapy to achieve CR1	
1	5
>1	6
MRD at transplantation	
Y	8
N	3
From diagnosis to transplant, mo	
≤6	6
>6-12	5
Donor type,n,	
HLA-identical sibling	3
10/10 matched unrelated	2
Related haploidentical	6
Donor sex, n,	
Female	6
Male	5
Donor/patient sex	
Female/male	4
Other combinations	7
Chromosome Type *de novo*	
Normal	6
46,XX,del(5)(q31(q35),del13(q12q14)	1
46,XX,add(16)(p13)	1
N/A	3

N/A, Not Available; ALL, Acute Lymphoblastic Leukemia; AML, Acute Myeloid Leukemia; MRD, Minimal Residual Disease.

**Table 2:** Characteristics of patients at transplant and prognosis.

Patient No.	Sex/Age(Y)	Diagnosis	White blood cell count *de novo*(×10^9^/L	Chromosome type *de novo*	Concurrent gene mutations	Donor type/Donor Sex	Months from diagnosis to transplant	GVHD prophylaxis	Conditioning regimen	MNC(×10^8^/kg)	CD34+cell (×10^6^/kg)	aGVHD	cGVHD	Relapse(days post transplant)	Follow-up(month)
1	F/37	AML	50	46,XX[20]	KRAS exon2 mutation,CCND3 exon5 mutation	Haplo/M	7	Tacrolimus+MTX+MMF	Bu+Flu+IDA+Cy+ATG	12	2.28	N	Y	Y(281)	DOD (25.7)
2	M/34	AML	1.63	46,XY[20]	JAK3 exon 15 mutation,TP53 exon8 mutation	MSD/M	8	CsA+MTX+MMF	Bu+Cy+Flu+Ara-C	18.98	6.83	Y(II)	N/A	Y(90)	DOD (10.8)
3	M/29	AML	N/A	N/A	JAK3 mutation,SH2B3 mutation,KDM6A mutation,PHF6 mutation	URD/M	6	Tacrolimus+MTX+MMF	TBI+Cy+Flu+Ara-C+ATG	13	4.03	Y(II)	Y	Y(89)	Alive(10.5)
4	M/43	T-ALL	47.63	46,XY[5]	FLT3-ITD mutation,BIRC3 exon2 mutation,SUZ12 exon8 mutation,PHF6 exon6 mutation,ASXL2 exon12 mutation	MSD/F	6	Tacrolimus+MTX	TBI+Cy+Flu+Ara-C+ATG	10	2.9	N	N	Y(456)	DOD (17.8)
5	M/31	T-ALL	26	46,XY[20]	SETBP1 EXON4,CREBBP EXON 19,EZH2 EXON 18	MSD/F	6	Tacrolimus+MTX	TBI+Cy+Flu+Ara-C	8.41	4.12	Y(IV)	Y	Y(1436)	DOD (53.2)
6	F/30	T-ALL	9.74	46,XX[2]	N/A	URD/F	6	CsA+MTX+MMF	TBI+Cy+Flu+Ara-C+ATG	10.52	4.2	Y(I)	N	Y(42)	DOD (2.6)
7	M/25	AML	12.26	N/A	N/A	Haplo/F	6	CsA+MTX+MMF	Bu+Clad+Ara-C+Cy+ATG	16.54	2.64	Y(I)	N	N	Alive(18.3)
8	M/23	AML	1.81	N/A	ETV6 mutation,PHF6 mutation,RUNX1 mutation	Haplo/M	8	Tacrolimus+MTX+MMF	Bu+Flu+IDA+Cy+ATG	13.74	2.41	Y(III)	N	N	TRM(8.1)
9	F/20	T-ALL	12.2	46,XX,del(5)(q31q35),del 13(q12q14)	N/A	Haplo/M	7	Tacrolimus+MTX+MMF	TBI+VP16+Cy+ATG	8.27	2.65	N	N	N	Alive(24.1)
10	F/29	T-ALL	223	46,XX,add(16)(p13)[3]/46,XX[3]	JAK1 exon19 mutation, NOTCH1 exon26 mutation, JAK3 exon13 mutation	Haplo/F	7	CsA+MTX+MMF	TBI+Cy+Flu+Ara-C+ATG	11.6	3.44	Y(I)	Y	N	Alive(7.0)
11	F/17	T-ALL	8.88	46,XY	JAK3 exon15 mutation,NOTCH1 exon34 mutation, WT1 exon7 mutation	Haplo/F	6	CsA+MTX+MMF	TBI+Cy+Flu+Ara-C+ATG	14.14	4.16	N	Y	N	Alive(27.1)

Y, yes; N, No; N/A, not applicable; F, Female; M, Male; AML, Acute Myeloid Leukemia; T-ALL, Acute T lymphocyte Leukemia; Haplo, Related Haploidentical Donor; MSD, Matched Sibling Donor; URD, matched Unrelated Donor; MTX, Methotrexate; CsA, Cyclosporin; MMF, Mycophenolate Mofetil; TBI, Total body irradiation; Bu, Busulfan; Flu, Fludarabine; Ara-C, Cytarabine; Cy, Cyclophosphamide; ATG, anti-thymocyte globulin; MNC, mononuclear cells.

### Transplant procedures

All 11 patients received myeloablative conditioning and allogeneic stem cell transplantation. The conditioning regimens for patients with AML were as follows: four patients with AML received conditioning regimens consisting of Busulfan (Bu, 3.2 mg/kg, days -9 to -7), Cyclophosphamide (Cy, 40 mg/kg, days -3 to -2), and Cytarabine(Ara-C, 2 g/m^2^, days -6 to -4) or Idarubicin (IDA, 10mg/kg, days -6 to -4) and Fludarabine (Flu, 30 mg/m^2^, days -6 to -4), or Cladribine (Clad, 5mg/m^2^, days -6 to -4). One patient with AML received TBI instead of Bu. Patients with T-ALL mostly received conditioning regimens as follows: TBI (3.3 Gy, days −9 to −7), Cy (40mg/kg, days −6, −5), Flu (30 mg/m^2^, days −4 to −2), and Ara-C (2 g/m^2^, days −4 to −2) as previously described (n=5) (7). One patient with T-ALL received etoposide instead of fludarabine and cytarabine. Patients who received haploidentical donor and matched unrelated donor HSCT received an additional 2.5mg/kg/day anti-T lymphocyte globulin (ATG) on days -5 to -2 (n=9). ATG was also given to the patient who was older than 40 years old (n=1). Calcineurin inhibitors plus short-term methotrexate along with or without mycophenolate mofetil were used for acute graft versus host disease prophylaxis as previously described (7, 8). Three patients received stem cells from matched-sibling donors, six from related haploidentical donors, and two from matched unrelated donors. More details are shown in **Table 2**.

### Statistical analysis

The primary endpoint was leukemia-free survival (LFS). Relapse or death were considered events. Overall survival (OS) was defined as the duration from stem cell administration to the last follow-up or death due to any cause. Transplant-related mortality (TRM) was defined as death in complete remission of leukemia after HSCT. Relapse was defined as any kind of morphological, cytogenetic, molecular disease recurrence, or extramedullary relapse. Minimal residual disease (MRD) was defined as any kind of molecular disease present without hematological relapse as follows: 1. Positive detection by real-time PCR of the SET-NUP214 fusion gene (ABL copies>10^4^, target gene/ABL >0%), 2. 0%<morphological leukemia blast cells <5%, and 3. leukemia cells/mononuclear cells >0% by flow cytometry analysis (capture 500,000 total events). Neutrophil engraftment was defined as the first date of neutrophil count ≥0.5×10^9^/L for three consecutive days. Platelet engraftment was defined as the first date of platelet count ≥20×10^9^/L and sustained for seven consecutive days independent of transfusion. Acute graft-versus-host disease (GVHD) was based on a previous standard (9). All dates were calculated from the first day of stem cell infusion to the day of the event or censored at the last follow-up. The Kaplan–Meier curve was calculated using SPSS 22.0. *P*<0.05 were considered statistically significant.

## Results

### Patient, disease, and transplant characteristics

The SET-NUP214 fusion gene could be detected in both T-ALL and AML patients in our single center. Normal chromosome phenotypes were mostly seen in these patients (n=6) (**Table 1**). JAK mutation (n=4) and PHF6 mutation (n=3) were mostly observed in these patients as concurrent mutations. Of all the patients with AML, three were categorized as AML without maturation. One patient was categorized as acute monocytic leukemia, and one was not otherwise specified de novo, according to the fifth edition of the WHO classification. One patient with T-ALL was categorized as ETP-ALL. Of all the 11 patients with acute leukemia, only 1 patient achieved complete molecular remission after one cycle of chemotherapy, indicating that this cohort of patients is somehow resistant to chemotherapy and should take HSCT as a treatment option, which is consistent with a previous report (5). All the patients underwent alloHSCT in CR1 and received peripheral stem cell infusion with a median positive CD34 count of 3.44 (range 2.28-6.82) ×10^6^/kg and median mononuclear cell count of 12.00 (range 8.27-18.98) ×10^8^/kg. Median times of neutrophil and platelet recovery were 14 days (range 11-17 days) and 15 days (range 11-33 days), respectively. All the patients achieved hematopoietic engraftment and complete remission at the molecular level after alloHSCT.

### OS and LFS

**Figure 1:**
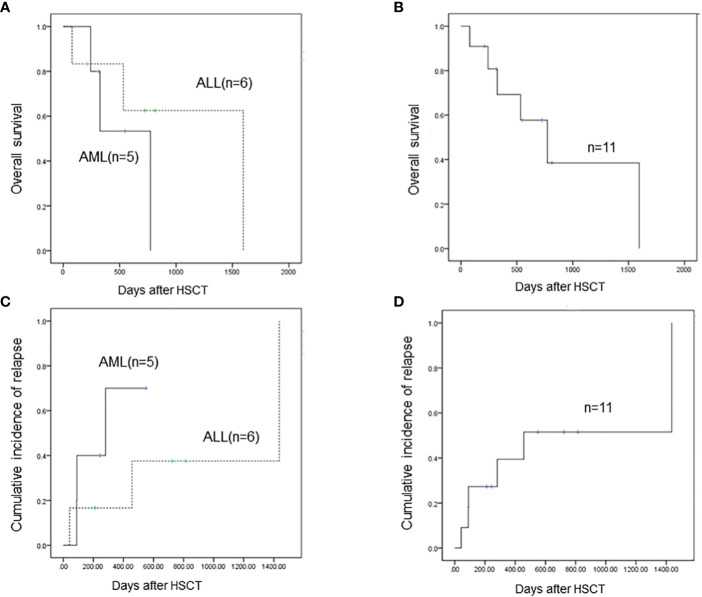
**(A)** The estimated 1-year overall survival (OS) is 53.3% for AML patients with SET-NUP214 fusion and the 3-year OS is 0%. The estimated 1-year OS for T-ALL patients with SET-NUP214 fusion is 83.3% and the 3-year OS is 62.5%. There are no statistical differences between the two groups (p=0.676). **(B)** The estimated 1-year and 3-year OS for all these 11 patients are 69.3% and 38.5%, respectively **(C)** Three of five (60%) patients with AML relapsed post-HSCT, and three of six patients with T-ALL (50%) relapsed post-HSCT. **(D)** For all 11 patients, the 1-year and 3-year cumulative incidence of relapse is 39.4% and 51.5%, respectively.

The estimated 1-year overall survival (OS) was 53.3% for patients with AML and the 3-year OS was 0% (**Figure 1A**). The estimated 1-year OS for patients with T-ALL was 83.3% and the 3-year OS was 62.5% (**Figure 1A**). There were no statistical differences between the two groups (p=0.676). The estimated 1-year and 3-year OS for all these 11 patients were 69.3% and 38.5%, respectively (**Figure 1B**). The 1-year and 3-year leukemia-free survival (LFS) for all patients were 69.3% and 38.5%, respectively. Of note, only one patient with T-ALL received regular chidamide as maintenance chemotherapy post-HSCT, and none of the others received any kind of maintenance chemotherapy post-HSCT. The patient taking chidamide was in LFS 814 days post-HSCT to the last follow-up.

### Relapse incidence and non-relapse mortality

In total, six patients relapsed after HSCT and five patients died of leukemia at the last follow-up. Three out of five (60%) patients with AML relapsed post-HSCT (**Figure 1C**): one patient had extramedullary involvement, one patient relapsed in bone marrow, and both patients died due to leukemia. One patient relapsed presenting as MRD and received combined chemotherapy including venetoclax and donor lymphocyte infusion; the patient achieved CR and was alive with chronic graft-versus-host disease to the last follow-up, which was 226 days after relapse post-HSCT. This patient remains the only one who survived after relapse post-HSCT. Three out of six patients with T-ALL (50%) relapsed post-HSCT and died of leukemia (**Figure 1C**). Two patients received intensive chemotherapy and subsequent DLI but did not respond to treatment. One patient chose palliative care and died 1 month after relapse. One patient with AML died of infection and was the only patient who died of transplant in this cohort. The TRM rate was 9.1%. Of note, the only patient who achieved molecular complete remission after one cycle of chemotherapy was in leukemia-free survival 211 days post-HSCT to the last follow-up. The six patients who relapsed post-HSCT never reached negative detection of the SET-NUP214 fusion gene before HSCT. In patients who did not relapse post-HSCT to the last follow-up, three out of five reached molecular remission before HSCT, and the number of cycles of chemotherapy to achieve negative fusion gene detection was 1,3, and 4, respectively. This indicates that patients with molecular remission before HSCT may achieve long survival after HSCT. Patients who never reach molecular remission have a high incidence of relapse rate (75%) post-HSCT but may still respond to HSCT. Thus, alloHSCT could be a salvage treatment option for these patients who are resistant to conventional chemotherapy. For all 11 patients, the 1-year and 3-year cumulative incidence of relapse was 39.4% and 51.5%, respectively (**Figure 1D**).

## Discussion

The NUP214 mapping at chromosome 9q34 has been reported as significant to genes in leukemogenesis (10). SET was reported as an oncogene that plays a role in transcription by modulating chromatin organization (11). The SET-NUP214, also known as TAF-1-CAN and SET-CAN, as a gene fusion has previously been described as a result of a chromosomal translocation t (9;9)(q34;q34) and del(9)(q34.11q34.13) (2–4). The fusion gene regulated leukemogenesis partly by upregulating the HOXA gene (2, 6). This fusion can be found in patients with T-ALL. The incidence of this fusion in patients with T-ALL is 4.6%-6%, according to data from different centers (5, 6). SET-NUP214 fusion has also been reported in cell lines of AML and single clinical cases of AML and AUL (1, 12, 13). In our study, AML with SET-NUP214 fusion was mostly present as AML without maturation, which may lead to poor survival even after alloHSCT. In patients with T-ALL, SET-NUP214 was reported to be strongly associated with corticosteroid and chemotherapy resistance but did not negatively influence clinical outcomes after HSCT (5). It is indicated that mutations of PHF6 and JAK1 are associated with the rearrangement of SET-NUP214 in T-ALL. In our cohort, concurrent mutations including JAK (n=4) and PHF6 (n=3) were mostly observed in both T-ALL and AML patients. Of note, JAK3 (n=3) and PHF6 (n=2) mutations were also observed in patients with AML. Regarding the prognosis of T-ALL patients with SET-NUP214, a Korean study showed that among four adult patients with T-ALL who presented with the fusion gene, only one patient who underwent HSCT survived (4). Song Y reported that in 17 AML and T-ALL patients with SET-NUP214, the median OS of 6 patients in chemotherapy was 10.5 (3-41) months, indicating none of the patients could survive without further alloHSCT. The OS and relapse-free survival of patients who underwent alloHSCT were better than those of the chemotherapy group (p=0.038) (14). In another study enrolling 11 T-ALL patients with SET-NUP214 fusion, the LFS and OS at 3 years of SET-NUP214–positive patients were 45% and 73%, respectively (5), which is somehow consistent with the prognosis of patients with T-ALL in our center. The present study and previous research all show disappointing LFS of these patients. The two patients with AML who were alive at the last follow-up did not exceed 3 years post-HSCT, thus we have no patients with AML who survived more than 3 years post-HSCT. However, regarding the high incidence of relapse, AML patients with SET-NUP214 fusion have a very poor prognosis even after alloHSCT. Bcl2 inhibitors such as venetoclax may be effective in patients with AML, as shown in our research but there is a need for further clinical trials with larger samples.

Recent research from Oka M linked NUP214 and NUP98, demonstrating that these two fusion proteins share some characteristics, including their nuclear bodies co-localized with CRM1 (also known as XPO1), and are both associated with aberrant activation of HOX genes. In addition, they are both physically and functionally associated with MLL1, which is also known as KMT2A (15). This suggests that treatment options for NUP98 rearranged acute myeloid leukemia may be adaptable to NUP214 rearranged acute myeloid leukemia patients but further evidence is needed. For patients with T-ALL, our previous research shows that maintenance of chidamide after alloHSCT did not significantly reduce the 1-year CIR of high-risk T-ALL but may improve the event-free survival (16). The one patient taking chidamide in our study is somehow in LFS to the last follow-up; there is a need for further investigation of the impact of chidamide on SET-NUP214 positive T-ALL.

Our research shows a very poor prognosis of acute leukemia patients with SET-NUP214 fusion even after alloHSCT. Despite all these patients undergoing alloHSCT in CR1 and achieving molecular remission shortly after HSCT, the major cause of death for these patients is still leukemia relapse. With very limited cases in our study, it is highly recommended that physicians should consider novel treatment strategies for these patients, including a stronger conditioning regimen and proper maintenance chemotherapy. Physicians should be alert of the high incidence of relapse for acute leukemia patients with SET-NUP214, even after alloHSCT. There is an urgent need for more clinical trials or research with larger samples for this group of patients.

## Data availability statement

The original contributions presented in the study are included in the article/supplementary material. Further inquiries can be directed to the corresponding author.

## Ethics statement

The studies involving humans were approved by Institute of Hematology and Blood Diseases Hospital, Chinese Academy of Medical Sciences. The studies were conducted in accordance with the local legislation and institutional requirements. Written informed consent for participation was not required from the participants or the participants’ legal guardians/next of kin in accordance with the national legislation and institutional requirements.

## Author contributions

YS: Conceptualization, Data curation, Writing – original draft. DY: Validation, Writing – review & editing. RZ: Validation, Writing – review & editing. XC: Validation, Writing – review & editing. QM: Validation, Writing – review & editing. JW: Validation, Writing – review & editing. WZ: Validation, Writing – review & editing. AP: Validation, Writing – review & editing. YH: Validation, Writing – review & editing. EJ: Supervision, Validation, Writing – review & editing. SF: Conceptualization, Funding acquisition, Supervision, Validation, Writing – review & editing.

## References

[1] von Lindern M Breems D van Baal S Adriaansen H Grosveld G. Characterization of the translocation breakpoint sequences of two DEK-CAN fusion genes present in t(6;9) acute myeloid leukemia and a SET-CAN fusion gene found in a case of acute undifferentiated leukemia. Genes Chromosomes Cancer (1992) 5:227e34. doi: 10.1002/gcc.2870050309 1384675

[2] Van Vlierberghe P van Grotel M Tchinda J Lee C Beverloo HB van der Spek PJ. The recurrent SET-NUP214 fusion as a new HOXA activation mechanism in pediatric T-cell acute lymphoblastic leukemia. Blood (2008) 111:4668e80. doi: 10.1182/blood-2007-09-111872 18299449 PMC2343598

[3] Lee SG Park TS Cho SY Lim G Park GJ Oh SH. T-cell acute lymphoblastic leukemia associated with complex karyotype and SET-NUP214 rearrangement: a case study and review of the literature. Ann Clin Lab Sci (2011) 41(3):267–72. 22075511

[4] Chae H Lim J Kim M Park J Kim Y Han K. Phenotypic and genetic characterization of adult T-cell acute lymphoblastic leukemia with del(9)(q34);SET-NUP214 rearrangement. Ann Hematol (2012) 91(2):193–201. doi: 10.1007/s00277-011-1289-x 21720744

[5] Ben Abdelali R Roggy A Leguay T Cieslak A Renneville A Touzart A. SET-NUP214 is a recurrent γδ lineage-specific fusion transcript associated with corticosteroid/chemotherapy resistance in adult T-ALL. Blood 123(12):1860–3. doi: 10.1182/blood-2013-08-521518 24449214

[6] Prokopiou C Koumas S Neokleous N Seimeni O Barmpouti A. SET-NUP214 rearrangement in isolation is insufficient to induce leukemia: a single center experience. Leuk Lymphoma (2016) 57(2):451–2. doi: 10.3109/10428194.2015.1049169 25956045

[7] Lv M Liu L He Y Yang D Ma Q Pang A. Outcomes of allogeneic or autologous stem cell transplantation followed by maintenance chemotherapy in adult patient with B-ALL in CR1 with no detectable minimal residual disease. Br J Haematol (2023) 202(2):369–378. doi: 10.1111/bjh.18846 37157187

[8] Cao YG He Y Zhang SD Liu ZX Zhai WH Ma QL. Conditioning regimen of 5-day decitabine administration for allogeneic stem cell transplantation in patients with myelodysplastic syndrome and myeloproliferative neoplasms. Biol Blood Marrow Transplant (2020) 26(2):285–91. doi: 10.1016/j.bbmt.2019.09.001 31494229

[9] Przepiorka D Weisdorf D Martin P Klingemann HG Beatty P Hows J. 1994 Consensus conference on acute GVHD grading. Bone Marrow Transplant (1995) 15(6):825–8. 7581076

[10] Xu S Powers MA. Nuclear pore proteins and cancer. Semin Cell Dev Biol (2009) 20:620e30. doi: 10.1016/j.semcdb.2009.03.003 19577736 PMC2706781

[11] Seo SB McNamara P Heo S Turner A Lane WS Chakravarti D. Regulation of histone acetylation and transcription by INHAT, a human cellular complex containing the set oncoprotein. Cell (2001) 104:119e30. doi: 10.1016/S0092-8674(01)00196-9 11163245

[12] Zhang H Zhang L Li Y Gu H Wang X. SET-CAN fusion gene in acute leukemia and myeloid neoplasms: report of three cases and a literature review. Onco Targets Ther (2020) 13:7665–81. doi: 10.2147/OTT.S258365.eCollection PMC7423397 32821125

[13] Quentmeier H Schneider Björn Röhrs S Romani J Zaborski M Macleod RA. SET-NUP214 fusion in acute myeloid leukemia- and T-cell acute lymphoblastic leukemia-derived cell lines. J Hematol Oncol (2009) 2:3. doi: 10.1186/1756-8722-2-3 19166587 PMC2636835

[14] Song Y Gong X-Y Wei S-N Li QH Zhang GJ Wang Y. Clinical analysis of SET-NUP214 fusion gene positive patients with acute leukemia. Zhongguo Shi Yan Xue Ye Xue Za Zhi (2023) 31(2):352–7. doi: 10.19746/j.cnki.issn.1009-2137.2023.02.007 37096505

[15] Oka M Otani M Miyamoto Y Oshima R Adachi J Tomonaga T. Phase-separated nuclear bodies of nucleoporin fusions promote condensation of MLL1/CRM1 and rearrangement of 3D genome structure. Cell Rep (2023) 42(8):112884. doi: 10.1016/j.celrep.2023.112884 37516964

[16] Guo W Cao Y Liu J Zheng X Wang M Zheng Y. Chidamide maintenance therapy in high-risk T-ALL/T-LBL after allo-HSCT: a prospective, single-center, single-arm study. Bone Marrow Transplant (2023) 58(10):1163–1166. doi: 10.1038/s41409-023-02045-w 37474728

